# New genetic variant in the *SERPINC1* gene: hereditary Antithrombin deficiency case report, familial thrombosis and considerations on genetic counseling

**DOI:** 10.1186/s12881-020-01001-5

**Published:** 2020-04-06

**Authors:** Margarita E. Polyak, Elena V. Zaklyazminskaya

**Affiliations:** Medical Genetics Laboratory, Petrovsky National Research Centre of Surgery, Abricosovsky lane, 2, Moscow, 119991 Russian Federation

**Keywords:** Antithrombin deficiency, *SERPINC1*, Case report, New genetic variant, Thromboprophylaxis

## Abstract

**Background:**

Inherited deficiency of the antithrombin (hereditary antithrombin deficiency, AT deficiency, OMIM #613118) is a relatively rare (1:2000–3000) autosomal-dominant disorder with high risk of venous thromboembolism. Mutations in the serpin family C member 1 gene (*SERPINC1)* can lead to Quantitative (type I) and Qualitative (type II) types of antithrombin deficiency. We describe a new genetic variant in the *SERPINC1* gene and our approach to variant interpretation.

**Case presentation:**

We observed a 29 y.o. female proband with the episode of venous thrombosis at the age of 18 and family history of thrombosis. The antithrombin level in our patient was low, 44–48% (AT deficiency type I). A new genetic variant c.662G > C (p.W221S) in the *SERPINC1* gene was detected in proband and affected father but was absent in healthy sister. We used in silico tools to evaluate the possible impact of p.W221S variant on protein structure and function. In mutated SERPINC1 protein a new N-linked glycosylation site is formed, however, it is unclear if the glycosylation at 219–221 site is possible.

**Conclusion:**

The proband was provided with appropriate genetic counseling and referred to a hematologist. Based on all the evidence we classify the p.W221S variant as variant of unknown clinical significance. In this paper we discuss some aspects of genetic counseling, variant interpretation and thromboembolic prophilaxis.

## Background

Antithrombin (AT) is a plasmatic inhibitor that inactivates coagulation factors. The primary target of AT is thrombin; however it also participates in Xa, IXa, XIa, and XIIa inhibition [1]. AT protein is encoded by the *SERPINC1* (serpin family C member 1) gene and consists of 432 amino acids, contains three disulfide bonds and four possible glucosylation sites. Two isoforms of AT, α and β, are known with considerable predomination of α-AT.

For the first time, the inherited AT deficiency as a thromboembolic risk factor was described in 1965 [2]. It was shown that inherited AT deficiency causes a 20-fold increase in incidence of venous thromboembolism (VTE) [3]. Inherited deficiency of antithrombin is a relatively rare (1 in 2000–3000 individuals) [4] disorder with autosomal dominant inheritance, caused by heterozygous mutations in *SERPINC1* gene. Homozygosity is extremely rare and incompatible with life [4]. Plasmatic levels of AT are usually less than 50% of normal values in most of the affected individuals. Thrombosis usually occurs by the age of 20 years old and the vast majority of patients become symptomatic by the fourth to fifth decades of life. Thrombosis might be induced by traumatisms, surgical interventions or other factors, and affects the venous system. Only a few cases of arterial thrombosis were reported [5].

Thus far, at least 220 mutations have been described in the *SERPINC1* gene. Mutations in the *SERPINC1* gene can lead to Quantitative (type I) and Qualitative (type II) types of AT deficiency by different molecular mechanisms. Usually the mutations causing haplo-insufficiency lead to AT type I deficiency. Many of the known missense mutations and in-frame deletions and insertions may lead to AT type II deficiency with normal levels of dysfunctional protein [6].

It is crucial to determine the exact cause of VTE for correct administration of medical treatment. It was estimated that pregnant women have a four- to five-fold increased risk of thromboembolism making total risk of VTE of 0.5–2.0 per 1000 women [7]. Thrombophilia and a history of VTE are considered to be the most important risk factors for thromboembolic complications during pregnancy and/or postpartum. This equates to determining the clinical significance of detected variants and to raising the efficiency of genetic counseling.

In this communication we report a new genetic variant detected in a family with AT deficiency. Our case illustrates the difficulties faced by genetic laboratories and genetic counselors when dealing with rare diseases in small families.

## Case presentation

**Fig. 1:**
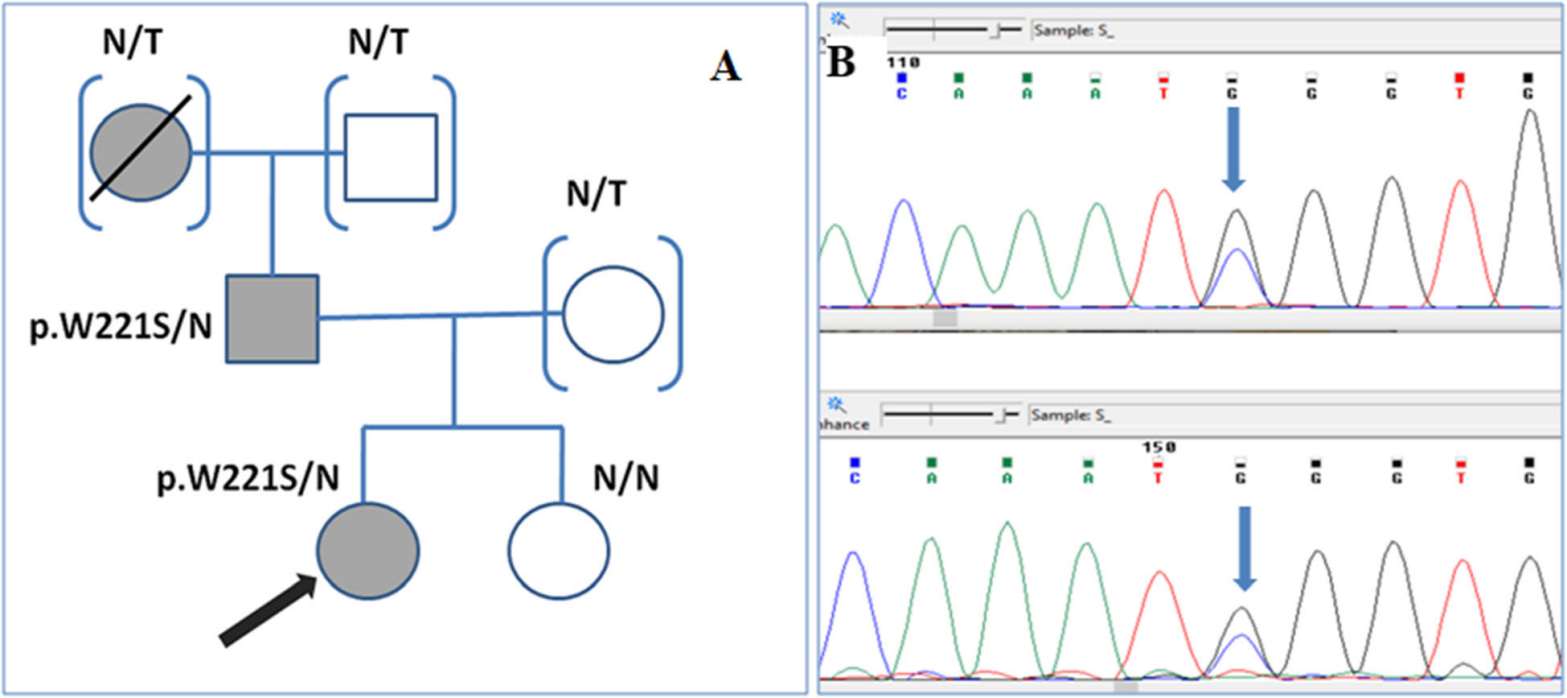
**a**. Family tree of the proband (marked with the red arrow); N/T – family members, not tested for p.W221S variant; N/N – family member without p.W221S variant (both allelеs are normal); blue shape filled – family members with the history of thrombosis. B. The fragment of chromatogram showing the sequence of exon 4 of *SERPINC1* gene in proband. Sequences made with forward primer (upper picture) and reverse-complement reverse primer (lower picture) are shown. The genetic variant c.662 G > C is marked with blue arrow

A 29 years old female proband experienced an episode of venous thrombosis at the age of 18. The thrombosis of left deep femoral vein occurred at the background of oral contraceptives. She also had a family history of thrombosis - father and grandmother had had thromboembolic events (Fig. 1). The proband was diagnosed with primary AT deficiency type I based on coagulation profile. Her main concern was to determine the hereditary nature of AT deficiency; she was highly interested in prospective and reproductive genetic counseling and planning for pregnancy. No DNA testing had been performed prior to our counseling.

The proband’s phenotype was unremarkable, no findings specific for syndromic or metabolic diseases were made. The level of antithrombin was measured routinely by colorimetric method (CS-5100i, Sysmex, “Berichrom Antithrombin III” kit). Mutation screening in the coding and adjacent intronic areas of the *SERPINC1* gene was performed by PCR-based bi-directional Sanger sequencing: oligoprimer design was performed in the Laboratory of medical genetics with PrimerQuest and NCBI Primer Blast tools; Sanger sequencing was performed on Applied Biosystems™ 3730xl DNA Analyzer, followed by sequence analysis with Chromas DNA Sequencing Software. Bioinformatic resources PolyPhen2, SIFT, Mutation Taster, NetGene2, and NetNGlyc 1.0 Server were used for in silico analysis of genetic variants. Databases “1000 Genomes”, “dbSNP”, and “Exome Variant Server” were used to evaluate the frequency of detected variant. Assessment of clinical significance of the detected variant was evaluated according to Joint Consensus Recommendation of the American College of Medical Genetics and Genomics and the Association for Molecular Pathology [8]. SWISS-MODEL integrated service was used for SERPINC1-W221S protein modeling [9–12]. We also used NGlycPred Server [13] to evaluate the probability of glycosylation occurring at a newly created site.

In the proband the residual antithrombin level was found to be significantly decreased down to 44–48% (AT deficiency type I) in independent repetitive measurements. Repetitive measurements were performed in order to confirm the low level of antithrombin in our patient and to rule out the artificially low results. A new genetic variant c.662G > C (p.W221S) in 4 exon of the *SERPINC1* gene was detected in the proband. Cascade family screening was performed; genetic variant c.662G > C (p.W221S) was found in the symptomatic father of the proband and was absent in the healthy sister. Variant c.662G > C (p.W221S) had not previously described in the literature and was absent in the “1000 Genomes” and other available databases. PolyPhen2, Mutation Taster and SIFT resources characterize c.662 G > C (p.W221S) as probably damaging; no possible splicing effect was detected.

**Fig. 2:**
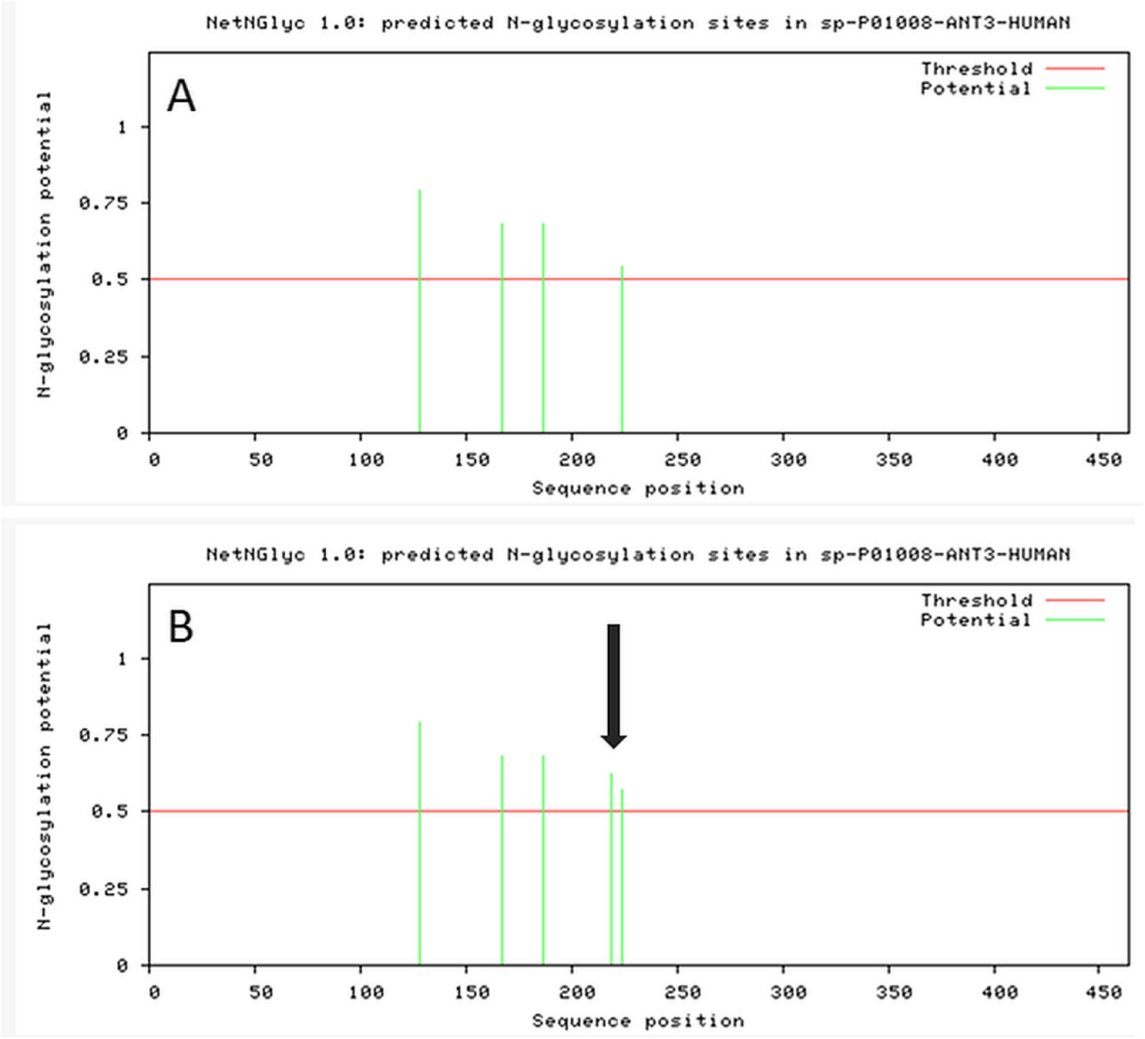
Glycosylation pattern of wild type SERPINC1 protein **a** and W221S- SERPINC1 protein **b**. Additional glycosylation site (predicted) is marked with arrow (prediction of NetNGlyc resource)

To improve the predictive value of the variant we proceeded with in silico analysis. Loss and acquisition of the glycosylation sites were both described in AT deficiency. Consequently, we analyzed glycosylation pattern in wt-AT and in W221S-AT using NetNGlyc 1.0 resource. According to NetNGlyc 1.0 Server prediction, p.W221S could create an additional glycosylation site (Fig. 2).

**Fig. 3:**
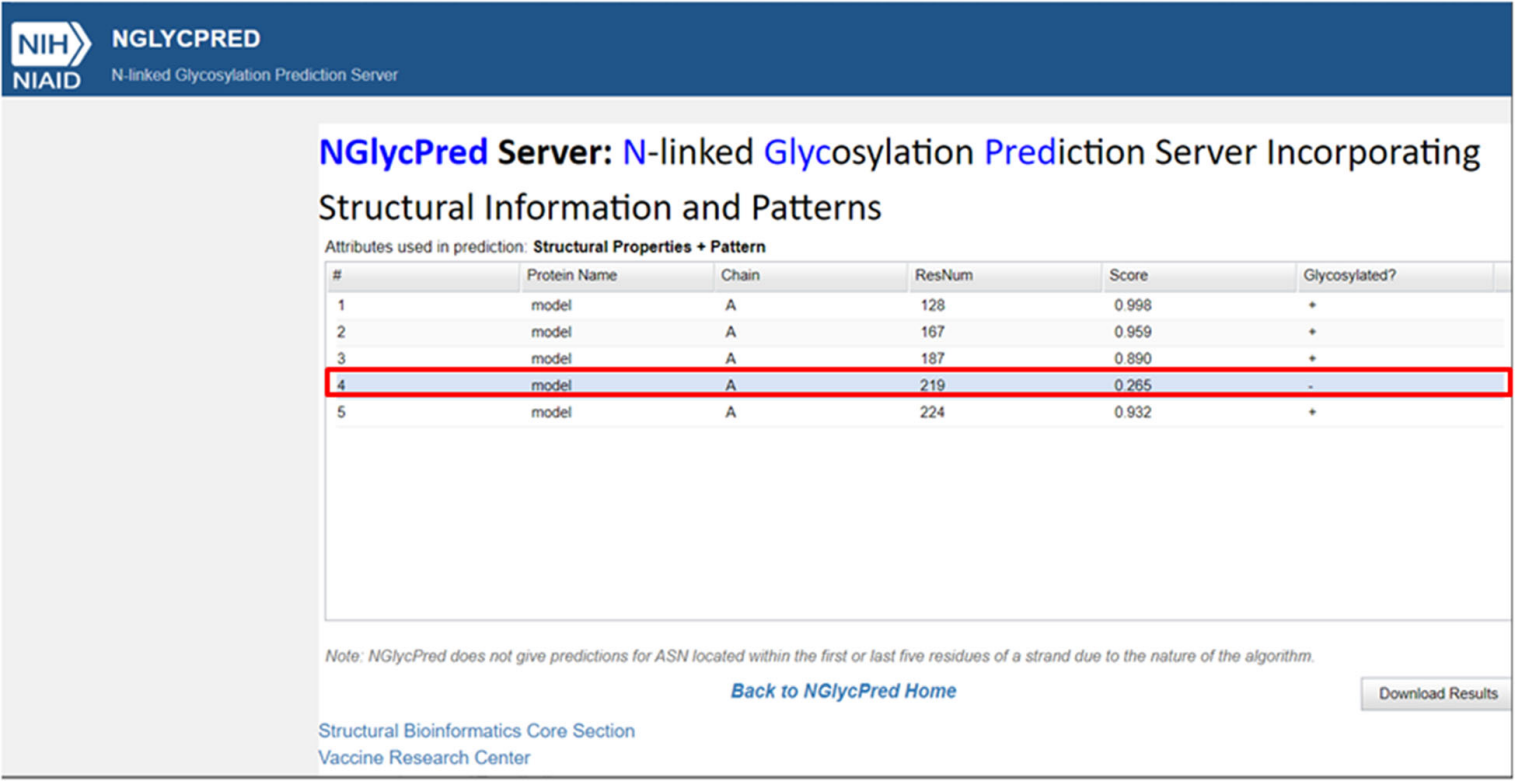
Prediction of NGlyPred Server. Additional glycosylation site 219–221 is predicted not to be glycosylated (boxed in red)

To evaluate the possibility of glycosylation occurring at a 219–221 site in the mutated SERPINC1 protein we built a model of it using a SWISS-MODEL service and analyzed it with NGlycPred Server. Both protein models with and without ligands were analyzed. According to NGlycPred Server prediction, the newly created 219–221 glycosylation site will not be glycosylated (Fig. 3).

Our classification of the c.662 G > C (p.W221S) variant was based on familial screening, population data from open-access databases (Exome Sequencing Project, 1000 Genomes, ExAC) and in silico analysis. We also assume that a PP4 criterion is applicable to our case, because clinical findings in our patient are specific for hereditary AT deficiency which is a monogenic disorder. No functional studies are available for this variant.

The proband was diagnosed with AT I deficiency, which is highly likely to be of hereditary nature. Based on all the evidence we classify the c.662 G > C (p.W221S) variant as a variant of unknown clinical significance.

## Discussion and conclusions

Glycosylation is one of the most common post-translational modifications of the proteins influencing functional activity [8]. Intracellular pathway of many proteins depends on the correct N-glycosylation.

**Fig. 4:**
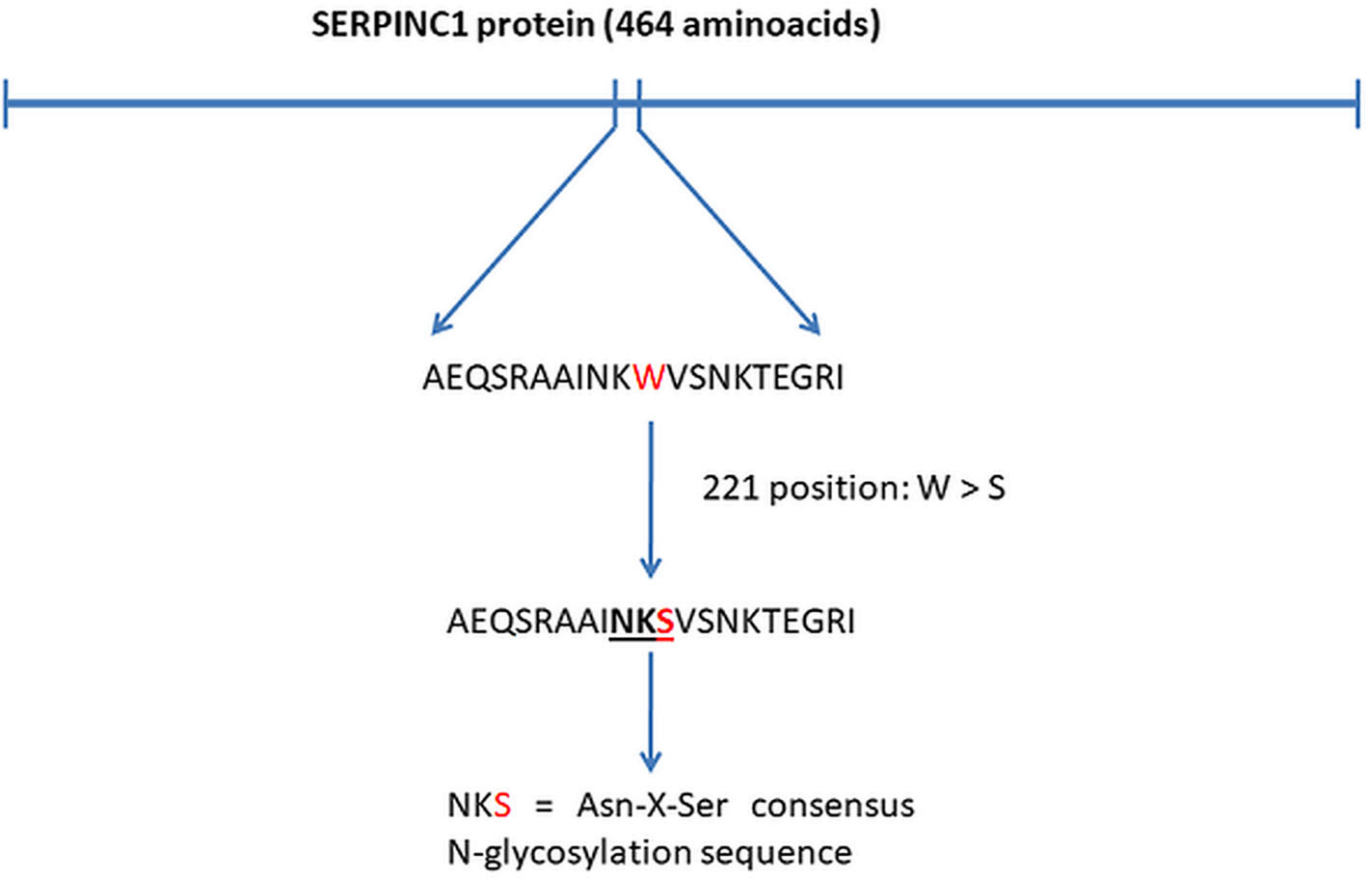
Creation of new possible glycosylation site in mutated SERPINC1 protein

Glycosylation disturbance is a known pathogenic mechanism in AT deficiency [14]. Consensus N-glycosylation site includes the following canonic amino acid sequence: Asn-X-Ser/Thr, or Asn-X-Cys, where “X” is any amino acid but proline. Genetic variant p.W221S creates a new site for N-linked glycosylation in the 219–221 amino acid position [Asn-Lys-Ser] sequence which is equivalent to Asn-X-Ser consensus N-glycosylation sequence (Fig. 4).

The disturbance of glycosylation patterns (either loss of glycosylation sites or formation of additional ones) was described in patients with AT deficiency. According to previous studies, the presence of additional glycosylation site results in increased weight of the protein and may alter folding and conformation [7]. Fitches et al. assume that mutated protein remains within the cells leading to deficiency in the bloodstream [14]. Consequently, in the heterozygous state only normal protein can reach the bloodstream. This corresponds to the clinical findings in our patient: she was diagnosed with type I AT deficiency because of persistently low levels of antithrombin in blood.

The patient was highly interested in periconception counseling so she was referred to a hematologist for evaluation of thromboprophylaxis options in her case. Pregnancy complications in women with coagulation disorders are being studied intensively. To our knowledge, recommendations on thromboprophylaxis in women with antithrombin deficiency during pregnancy are controversial. Guidelines of the American College of Chest Physicians were issued in 2012 [15]. The authors suggested that women with inherited thrombophilia and a history of pregnancy complications should not use special antithrombotic prophylaxis (Grade 2C). A recent study of James et al. (2017) [16] claims that the risk of pregnancy-associated VTE is about 3–17.7%. It is a wide risk range difficult for clinical implication. In the review by Rhéaume et al. the incidence of pregnancy associated venous thromboembolism was estimated to be of 11.6%, which is much higher than that in the general population (0.1%) [2], however, the data only from asymptomatic women with positive family history were included in this review. Overall, most of the studies have limitations because of small number of patients enrolled, but it is not unusual for rare genetic disorders.

It is possible that the lack of sustained recommendations is due to relative rareness of the disease. Some studies use an integrative approach to accumulate clinical [17] and genetic data. Gradually, new recommendations appear for individual genetic diseases (for example, for patients with channelopathies, Beckwith-Wiedemann syndrome, etc.). We assume that the accumulated experience of small, though numerous, studies and a request for targeted management of such groups of patients will lead to the appearance of recommendations on thromboprophylaxis in patients with AT-III deficiency, during pregnancy as well.

James et al. in their review of 2017 states that genetic testing in diagnosis of AT deficiency is worthy of consideration [16], however, they do not discuss it in the review. Our clinical case exemplifies the problems of genetic testing in rare disorders. Hereditary antithrombin deficiency is a monogenic disorder with no less than 220 pathogenic genetic [4] variants described in the *SERPINC1* gene. However, to our knowledge, only one hotspot was described in the *SERPINC1* gene: genetic variants c.881G > T (p.Arg294Leu) and c.883G > A (p.Val295Met) predominate in the Chinese cohort of VTE patients [18]. Also genetic variant p.Pro305His [19] was detected in several Indian families. No hotspot variants were described for Caucasian populations. Therefore, most of the genetic variants described are found within one family, making statistical analysis of prevalence of the variant in affected individuals unavailable. Functional studies have also been performed for a limited number of genetic variants so far, and the results of in silico analysis can be controversial.

**Fig. 5:**
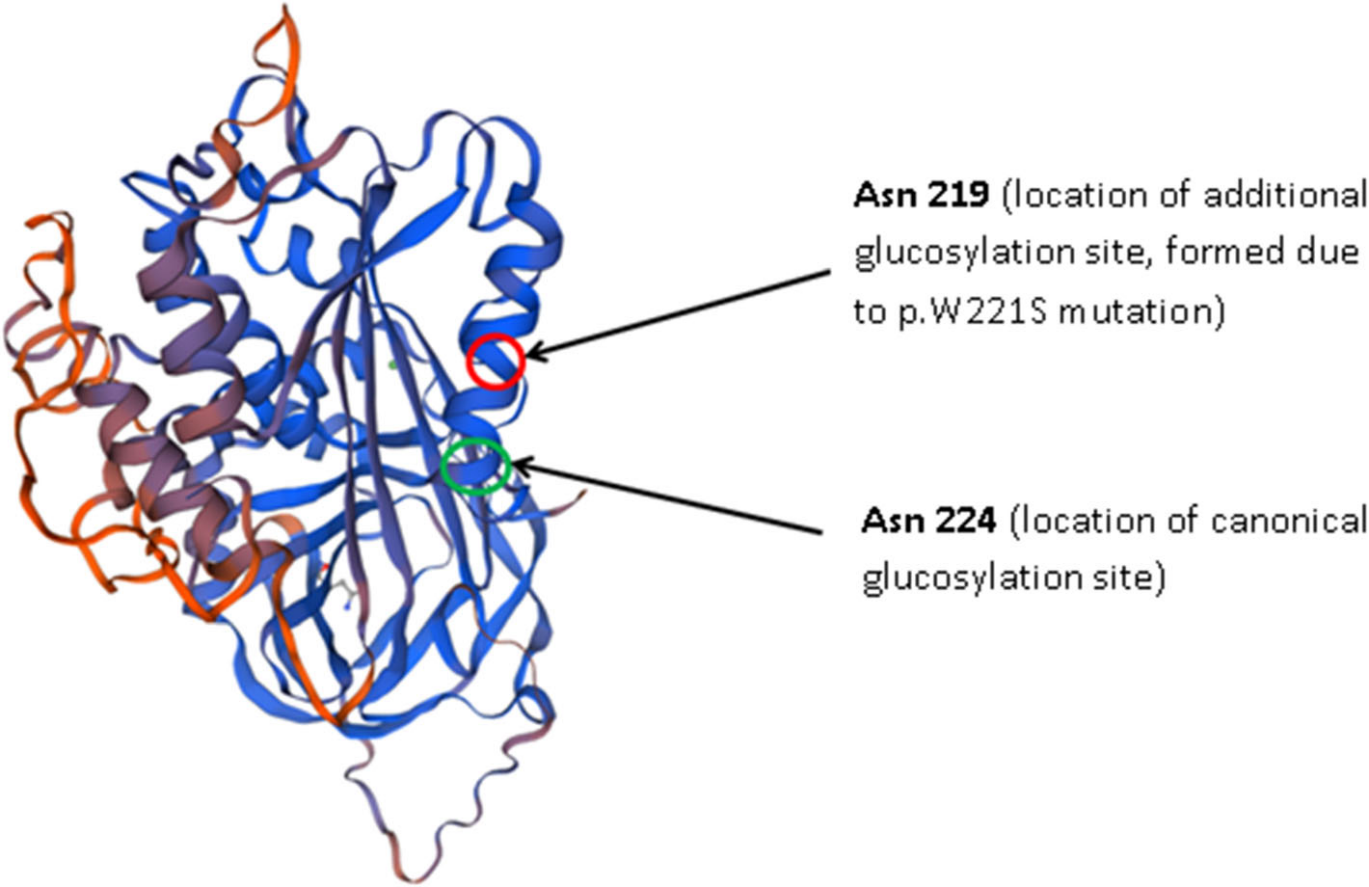
Structure of SERPINC1-W221S protein with locations of canonical and additional glycosylation sites (SWISS-MODEL)

We used several in silico tools to evaluate the possible impact of p.W221S variant on the structure and post-translational modification. However, the results were inconclusive. There is little doubt in the presence of additional glycosylation site at 219–221 aminoacid positions. It is possible though that its close location to the canonical 224–226 glycosylation site will not allow the glycosylation to occur due to steric hindrance (Fig. 5).

ACMG criteria impose strict requirements for a genetic variant characterization. A genetic variant will be considered likely pathogenic or pathogenic only if several criteria from different groups are met. On the one hand, it allows us to accumulate data and interpret it carefully. On the other hand, it can cause difficulties in routine genetic counseling for patients with rare genetic disorders. Genetic variant c.662 G > C (p.W221S) seems quite typical for antithrombin deficiency, as glycosylation altering genetic variants have been previously described [14]. Genetic variant p.S82N is a well described mutation leading to a new glycosylation site and a higher molecular weight product formation. As a matter of interest, the first gain-of-glycosylation genetic variant ever reported in the literature was a p.I7N mutation, detected in a patient with pulmonary embolism and AT deficiency [20]. However, in the absence of functional studies for p.W221S variant and a small number of proband’s relatives available for screening we can only classify it as variant of unknown clinical significance. Genetic counseling for the family will not be highly effective: we were not able to rule out AT deficiency in the asymptomatic proband’s sister based on genetic data only. In this context clinical evaluation is highly important. It is expected from hematologists to evaluate possible secondary causes of AT deficiency in a proband.

Bioinformatic analysis is highly useful for clinical laboratories with limited experimental facilities. However, functional studies are highly desirable to evaluate the possible effects of a genetic variation.

In our patient diagnosed with antithrombin deficiency we detected a new *SERPINC1* variant – c.662 G > C (p.W221S). According to in silico analysis the mutated protein carries a possible additional glycosylation site in the Asn219 position, however, it remains unclear, if the mutated site will be glycosylated. Additional glycosylation was previously described as a pathogenic mechanism in AT deficiency. The combination of bioinformatic analysis and functional studies will allow improving the efficiency of correct interpretation of rare genetic variants and, therefore, genetic counseling in patients with rare disorders.

## Data Availability

Authors confirm that all relevant data are included in the article. We reported the p.W221S variant in dbSNP/ClinVar database (rs1553218111, accession: VCV000369838.2, variant ID:369838). Data that supports the findings (e.g. sequence chromatograms of other exons of *SERPINC1* gene in proband and/or sequence chromatograms of exon 4 of *SERPINC1* gene in proband’s sister and father) are available from the corresponding author upon request.

## Abbreviations

ACMG: American College of Medical Genetics
AT: Antithrombin
*SERPINC1*: Serpin family C member 1
OMIM: Online Mendelian Inheritance in Man
PCR: Polymerase chain reaction
VTE: Venous thromboembolism
wt: Wild type
